# Impact of social-psychological factors on anxiety before gastrointestinal endoscopy and quality of life

**DOI:** 10.3389/fpsyt.2025.1624437

**Published:** 2025-08-20

**Authors:** Zhen-peng Huang, Kong-jin Quan, Bin-bin Wen, Jia-feng Lin, Tao Liu, Li-ping Yang, Li-ping Meng

**Affiliations:** ^1^ Faculty of Nursing, Guangxi University of Chinese Medicine, Nanning, China; ^2^ Department of Gastrointestinal Endoscopy, Guangxi International Zhuang Medical Hospital, Nanning, China

**Keywords:** social factors, psychological factors, anxiety, gastrointestinal endoscopy, quality of life

## Abstract

**Introduction:**

This study aimed to examine the impact of social-psychological factors on anxiety before gastrointestinal endoscopy and its effect on patients’ quality of life (QoL).

**Methods:**

Patients scheduled for gastrointestinal endoscopy were recruited for the study. Demographic characteristics, social factors, lifestyle information and endoscopy-related data were obtained through self-reports and the hospital information system. The 7-item Generalized Anxiety Disorder Questionnaire (GAD-7) was used to assess anxiety, while depression and somatization were evaluated using the Patient Health Questionnaire-9 (PHQ-9) and PHQ-15, respectively. Sleep quality was measured with the Pittsburgh Sleep Quality Index (PSQI), social support was assessed using the Social Support Rating Scale (SSRS), and QoL was evaluated through the 12-item Short Form Survey (SF-12).

**Results:**

The prevalence of anxiety before gastrointestinal endoscopy was 34.44%. Gender, age, sleep quality, social support, depression, and somatization were associated with anxiety (all *P*<0.05). Independent predictors of anxiety were gender, age, PSQI, SSRS, PHQ-9 and PHQ-15 scores (all *P*<0.05). Furthermore, PSQI, PHQ-9, and PHQ-15 scores were positively correlated with the severity of anxiety (all *P*<0.05). Patients with anxiety exhibited lower scores in domains of the SF-12, including general health (GH), physical functioning (PF), role-physical (RP), bodily pain (BP), role-emotional (RE), mental health (MH), vitality (VT), and social functioning (SF). Both physical component summary (PCS) and mental component summary (MCS) scores were reduced. Notably, GH, RP, RE, MH, VT, SF, and MCS scores were negatively correlated with varying levels of anxiety (all *P*<0.05).

**Conclusion:**

Social-psychological factors play a role in anxiety before gastrointestinal endoscopy; anxiety can negatively affect patients’ QoL.

## Introduction

Gastrointestinal endoscopy allows for direct visualization of the alimentary tract and remains one of the most effective methods for detecting, diagnosing, and treating gastroenterological diseases (1). Although recent advances, such as artificial intelligence-assisted endoscopy, have emerged rapidly, traditional gastrointestinal endoscopy continues to play a critical role in the detection of gastric and colorectal lesions. It is widely regarded as the gold standard for diagnosing gastric and colorectal cancer and precancerous conditions (2–4). Procedures such as gastroscopy and colonoscopy, although minimally invasive and generally considered safe and effective, are frequently associated with heightened patient anxiety (5, 6). The underlying contributions to this pre-procedural anxiety remain poorly understood. Importantly, anxiety prior to gastrointestinal endoscopy not only affects psychological well-being but may also influence the examination process, treatment compliance, and postoperative recovery (7, 8).

Quality of life (QoL) is a key measure in evaluating health status and effectiveness of clinical interventions (9). Recent research has demonstrated that symptoms of anxiety are negatively associated with Qol, with more severe anxiety corresponding to more significant Qol impairments (10, 11). Whether anxiety specifically related to gastrointestinal endoscopy similarly impacts QoL remains unclear. Therefore, understanding and addressing patients’ anxiety in the context of endoscopic procedures is of growing importance in both clinical and psychological care.

The Theory of Unpleasant Symptoms (TOUS), proposed in 1995, emphasizes that unpleasant symptoms are subjectively perceived experiences that reflect a disruption in an individual’s normal functioning. TOUS identifies three key categories of factors that influence the symptom experience: physiological, psychological, and environmental factors. Physiological factors pertain to bodily function and pathological conditions; psychological factors involve emotional states, coping mechanisms, and perceived uncertainty; and environmental factors encompass social contexts, including family dynamics and levels of social support, which shape how symptoms are perceived. Furthermore, unpleasant symptoms can lead to specific symptom outcomes, referring to the consequences or prognoses following the experience of these symptoms (12, 13). Our previous research has demonstrated that socio-psychological factors influence both physical and mental health outcomes (14–16).

Building on the TOUS, this study aimed to investigate the impact of socio-psychological factors on anxiety prior to gastrointestinal endoscopy, as well as their subsequent effect on patients’ QoL.

## Materials and methods

### Participants and study design

Patients who visited the Department of Gastrointestinal Endoscopy at the Affiliated Hospital of Guangxi University of Chinese Medicine and were scheduled to undergo gastrointestinal endoscopy were recruited using a snowball sampling strategy between July and December 2024.

The inclusion criteria were as follows: (1) patients scheduled to undergo gastrointestinal endoscopic procedures; (2) no prior history of mental or mood disorders; (3) without severe physical illness; (4) no prior use of anti-anxiety or antidepressant treatments; (5) no experience of major life events within the past three months; (6) without cognitive impairment; (7) ability to read and write in Chinese.

This study was approved by the Institutional Ethics Committee of the Affiliate Hospital of Guangxi University of Chinese Medicine (Approval No. KY 2024-198). All participants provided written informed consent prior to their inclusion in the study and participated voluntarily. All procedures were conducted in accordance with relevant ethical guidelines and regulations, including the Declaration of Helsinki.

### Sample size calculation

According to standard clinical research sample size estimation formulas, the average prevalence of anxiety prior to gastrointestinal endoscopy is approximately 50%. Considering an estimated 10% attrition rate, the minimum required sample size for this study was determined to be 424 participants (17, 18).

### Measures and questionnaires

Demographic features, including gender and age, were collected. Social features and lifestyle data were self-reported by all participants and included place of residence, educational level, marital status, smoking, and alcohol consumption.

Psychological and endoscopy-related data, including family history of digestive diseases, H pylori infection status, number of prior endoscopies, use of anesthesia during endoscopy, types of endoscopic procedures, and diagnoses from both gastroscopic and colonoscopy, were obtained through self-reports in the hospital information system.

The Chinese version of the 7-item Generalized Anxiety Disorder questionnaire (GAD-7) was used to assess whether participants experienced anxiety. Each item score was calculated, and a total score above 5 indicated the presence of anxiety. Scores between 5 and 9 indicated mild anxiety, 10 to 14 indicated moderate anxiety, and scores above 15 indicated severe anxiety. The Cronbach’s alpha for the GAD-7 in this study was 0.92 (19).

The Chinese version of the Patient Health Questionnaire-9 (PHQ-9) was used to screen for depression. Each item was scored and summed, with a total score above 5 indicating the presence of depression. Scores between 5 and 9 indicated mild depression, 10 to 14 indicated moderate depression, 15 to 19 indicated moderately severe depression, and scores above 20 indicated severe depression. The Cronbach’s alpha for the PHQ-9 was 0.88 (20).

The Chinese version of the Patient Health Questionnaire-15 (PHQ-15) was used to screen for somatization and assess the severity of somatic symptoms. Each item was scored, and the total score was used to determine symptom severity. A total score above 6 indicated the presence of somatization. Scores from 6 to 15 indicated mild somatization, scores from 16 to 25 indicated moderate somatization, and scores above 26 were classified as severe somatization (21). The Cronbach’s alpha for the PHQ-15 in this study was 0.79 (22).

Sleep quality was measured using the Chinese version of the Pittsburgh Sleep Quality Index (PSQI). The PSQI consists of 19 self-rated items and differentiates between good and poor sleep quality by evaluating seven components: subjective sleep quality, sleep latency, sleep duration, habitual sleep efficiency, sleep disturbances, use of sleeping medication, and daytime dysfunction over the previous month. A total PSQI score above 5 indicates poor sleep quality (23). The Cronbach’s alpha for the PSQI was 0.71, and the intraclass correlation coefficient was 0.90 (24).

Social support was assessed using the Social Support Rating Scale (SSRS), which evaluates three components: objective social support, subjective social support, and utilization of social support. Each item was scored, and total scores below 20 indicated low-level social support, scores between 20 and 29 indicated medium-level social support, and scores above 30 indicated high-level social support (25, 26).

The Chinese version of the 12-item Short Form Survey (SF-12) was used to measure QoL. The SF-12 is a self-reported questionnaire that includes eight domains and two summary components. The domains assessed by the SF-12 include general health (GH), physical functioning (PF), role-physical (RP), bodily pain (BP), role-emotional (RE), mental health (MH), vitality (VT), and social functioning (SF). The two summary components are the physical component summary (PCS) and the mental component summary (MCS), respectively (27). The reliability and validity of the Chinese version of the SF-12 have been confirmed and found to be comparable to those of the Chinese version of the SF-36 (28).

### Statistical analysis

A *post-hoc* power analysis was conducted to ensure statistical power for sample size calculation by G*power version 3.1. Statistical analyses were conducted using IBM SPSS Statistics version 25.0. Continuous variables were presented as mean ± standard deviation, and categorical variables were expressed as percentages. Associations between relevant factors and study outcomes were reported as odds ratios (OR) with 95% confidence intervals (95% *C.I.*). Continuous variables were compared using independent *t*-tests. Categorical variables were compared using the chi-square test or Fisher’s exact test as appropriate. Independent factors associated with anxiety prior to gastrointestinal endoscopy were analyzed using logistic regression analysis. *P*< 0.05 was considered statistically significant.

## Results

### Incidence of anxiety before gastrointestinal endoscopy

A total of 504 patients who underwent gastrointestinal endoscopy were enrolled in this study, of whom 482 completed the questionnaires (power is 0.9126). Among them, 236 (48.96%) were male and 246 (51.04%) were female. The participants’ age ranged from 18 to 80 years, with a mean age of 44.21 ± 13.751 years.

The overall prevalence of anxiety before gastrointestinal endoscopy was 34.44%. Among the patients who experienced anxiety prior to the procedure, 124 (74.70%) had mild anxiety, 30 (18.07%) had moderate anxiety, and 12 (7.23%) had severe anxiety.

### Social factors, lifestyles, and anxiety before gastrointestinal endoscopy

Various demographic and social factors, including gender, age, and level of social support, were found to significantly influence the incidence of anxiety before gastrointestinal endoscopy (all *P*<0.05). In addition, unhealthy lifestyle factors such as poor sleep quality were also associated with higher anxiety levels (*P*< 0.05). However, smoking and alcohol consumption did not show a significant association with anxiety (all *P*> 0.05) (**Table 1**).

**Table 1:** Demographic, social factors and lifestyles in anxiety before gastrointestinal endoscopy [*x* ± *s*/n(%)].

Variables	Category	Anxiety	Without anxiety	Statistical value
Gender	Male	68 (40.96)	169 (53.48)	
	Female	98 (50.04)	147 (46.52)	*x* ^2^ = 6.8224, *P*=0.009
Age	Below 30 years old	45 (27.11)	45 (14.24)	
	31–40 years	43 (25.90)	74 (23.42)	
	41–50 years	28 (16.87)	83 (26.27)	
	51–60 years	30 (18.07)	68 (21.52)	
	61–70 years	17 (10.24)	37 (11.70)	
	Above 71 years old	3 (1.81)	9 (2.85)	*x* ^2^ = 11.2842, *P*=0.0103
	Mean age	41.75 ± 14.337	45.49 ± 13.275	*t*=2.791, *P*=0.006
Place of residence	Urban area	237 (75.00)	120 (72.29)	
	Rural area	79 (25.00)	46 (27.71)	*x* ^2^ = 0.4164, *P*=0.5188
Educational level	High school or below	62 (37.35)	144 (45.57)	
	College	96 (57.83)	155 (15.82)	
	Graduate	8 (4.82)	17 (5.38)	*x* ^2^ = 3.6768, *P*=0.2985
Marital status	Single	52 (16.45)	29 (17.46)	
	Married	241 (76.26)	121 (65.76)	
	Divorced	23 (63.89)	16 (9.64)	*x* ^2^ = 0.9808, *P*=0.6124
Smoking		25 (15.06)	66 (20.89)	*x* ^2^ = 0.1212, *P*=0.7277
Alcohol		45 (27.11)	96 (30.38)	*x* ^2^ = 0.5627, *P*=0.4532
PSQI scores	Sleep quality	1.61 ± 0.752	1.22 ± 0.711	*t*=-5.608, *P*<0.001
	Sleep latency	1.66 ± 0.976	1.26 ± 0.951	*t*=-4.348, *P*=327.884
	Sleep duration	0.86 ± 0.934	0.76 ± 0.900	*t*=-1.118, *P*=0.265
	Habitual sleep efficiency	0.76 ± 0.900	0.61 ± 0.948	*t*=-1.564, *P*=0.119
	Sleep disturbance	1.36 ± 0.603	0.99 ± 0.497	*t*=-7.162, *P*<0.001
	Use of sleeping medication	0.14 ± 0.527	0.04 ± 0.273	*t*=-2.760, *P*=0.006
	Daytime dysfunction	1.93 ± 0.935	1.01 ± 0.971	*t*=-2.760, *P*=0.006
	Total scores	8.33 ± 3.468	5.89 ± 3.332	*t*=-10.175, *P*<0.001
Poor sleep quality		143 (86.14)	131 (41.46)	*x* ^2^ = 88.5987, *P*<0.001
SSRS scores	Objective support	16.09 ± 4.749	16.09 ± 4.749	*t*=5.565, *P*<0.001
	Subjective support	7.09 ± 2.785	7.65 ± 2.799	*t*=2.076, *P*=0.039
	Utilization of social support	3.99 ± 1.966	4.36 ± 2.167	*t*=1.909, *P*=0.057
	Total scores	24.70 ± 6.977	28.10 ± 7.179	*t*=5.037, *P*<0.001
Degree of social support	Poor	40 (24.10)	39 (12.34)	
	Moderate	93 (56.02)	165 (52.22)	
	Good	33 (19.88)	112 (35.44)	*x* ^2^ = 18.2323, *P*=0.001

PSQI, Pittsburgh Sleep Quality Index; SSRS, Social Support Rating Scale.

Independent t-tests is used analyzed for PSQI scores, SSRS scores; Chi-square test is used analyzed for gender, age, place of residence, educational level, marital status, smoking, alcohol, poor sleep quality and degree of social support.

### Psychological factors and anxiety before gastrointestinal endoscopy

This study confirmed that psychological factors, specifically depression and somatization, significantly affected the incidence of anxiety before gastrointestinal endoscopy (all P<0.05). Most patients who experienced anxiety were also found to suffer from mild depression and mild somatization (all *P*<0.05) (**Table 2**).

**Table 2:** Psychological factors in anxiety before gastrointestinal endoscopy [*x* ± *s*/n(%)].

Variables	Category	Anxiety	Without anxiety	Statistical value
PHQ-9 scores		6.48 ± 4.700	1.52 ± 2.243	*t*=-15.675, *P*<0.001
Degree of depression	Mild	68 (38.55)	20 (6.33)	
	Moderate	24 (14.46)	3 (0.95)	
	Moderate-severe	7 (4.22)	3 (0.95)	
	Severe	3 (1.81)	0 (0)	*x* ^2^ = 165.2221, *P*<0.001
PHQ-15 scores		10.27 ± 4.698	5.59 ± 3.863	*t*=-11.702, *P*<0.001
Degree of somatization	Mild	115 (69.28)	146 (46.20)	
	Moderate	25 (15.06)	3 (0.95)	
	Severe	0 (0)	0 (0)	*x* ^2^ = 85.5864, *P*<0.001

PHQ-9: Patient Health Questionnaire-9; PHQ-15: Patient Health Questionnaire-15.

Independent t-tests is used analyzed for PHQ-9 scores and PHQ-15 scores; Fisher’s exact test is used analyzed for degree of depression and somatization.

### Physiological and endoscopy-related factors and anxiety before gastrointestinal endoscopy

In this study, physiological and endoscopy-related factors, including family history of digestive diseases, H. pylori infection, the number of previous endoscopies, whether the procedure was performed under sedation, type of endoscopy performed, and the endoscopic diagnosis, were not significantly associated with anxiety before gastrointestinal endoscopy (all *P*>0.05) (**Table 3**).

**Table 3:** Physiological and endoscopic related factors in anxiety before gastrointestinal endoscopy [n(%)].

Variables	Category	Anxiety	Without anxiety	Statistical value
Family history	Peptic ulcer	11 (6.63)	14 (4.43)	
	Gastritis	38 (22.89)	69 (21.84)	
	Cancer	9 (5.42)	18 (5.70)	
	No family history	108 (65.06)	215 (68.03)	*x* ^2^ = 1.2253, *P*=0.7469
H pylori. infection	Infection not eradication	18 (10.84)	46 (14.56)	
	Infection and eradication	7 (4.22)	13 (4.11)	
	No infection	54 (32.53)	107 (33.86)	
	Unknown	87 (52.41)	150 (47.47)	*x* ^2^ = 1.7312, *P*=0.6300
Times for endoscopy	The first time	93 (56.02)	172 (54.43)	
	The second time	34 (20.48)	78 (24.68)	
	The third time	24 (14.46)	34 (10.76)	
	The fourth time and above	15 (9.04)	32 (10.13)	*x* ^2^ = 2.2468, *P*=0.5228
Endoscopy within sedative		152 (91.57)	284 (89.87)	*x* ^2^ = 0.3613, *P*=0.5478
Types of endoscopies examination	Gastroscope	68 (38.55)	20 (6.33)	
	Colonoscopy	24 (14.46)	3 (0.95)	
	Gastroscope and Colonoscopy	7 (4.22)	3 (0.95)	*x* ^2^ = 0.9135, *P*=0.6334
Gastroscopic diagnosis	No abnormalities	100 (64.10)	195 (66.78)	
	Peptic ulcer	16 (10.26)	24 (8.22)	
	Polyp	31 (19.87)	41 (14.04)	
	Cancer	3 (1.92)	6 (2.05)	
	Gastroesophageal reflux disease	6 (3.85)	23 (7.88)	
	Esophagogastric varices	0 (0)	3 (1.03)	*x* ^2^ = 5.7266, *P*=0.1257
Colonoscopic diagnosis	No abnormalities	56 (52.34)	104 (48.83)	
	Peptic ulcer	1 (0.93)	2 (0.94)	
	Polyp	41 (38.32)	89 (41.78)	
	Cancer	0 (0)	2 (0.94)	
	Inflammation	9 (8.41)	15 (7.04)	
	Anorectal varices	0 (0)	1 (0.47)	*x* ^2^ = 0.4566, *P*=0.7959

Chi-square test is used analyzed for family history, H pylori. infection, times for endoscopy and endoscopy within sedative and types of endoscopies examination. Fisher’s exact test is used analyzed for gastroscopic diagnosis and colonoscopic diagnosis.

### Relevant independent factors for anxiety before gastrointestinal endoscopy

Gender (OR=1.657, 95%C.I. 1.133-2.423), age (OR=0.967, 95%C.I. 0.952-0.983), PSQI scores (OR=1.258, 95%C.I. 1.180-1.342), SSRS scores (OR=0.951, 95%C.I. 0.923-0.980), PHQ-9 scores (OR=1.115, 95%C.I. 1.045-1.188) and PHQ-15 scores (OR=1.490, 95%C.I. 1.350-1.645) were all identified as independent factors associated with anxiety before gastrointestinal endoscopy (all *P*<0.05) (**Table 4**).

**Table 4:** Logistic regression analyses on selected factors associated with anxiety before gastrointestinal endoscopy.

Selected factors	B	S.E.	Wald	df	Sig.	Exp (B)	95%C.I. for Exp (B)
Gender	0.505	0.194	6.775	1	0.009	1.657	(1.133, 2.423)
Age	-0.033	0.008	16.921	1	<0.001	0.967	(0.952, 0.983)
PSQI scores	0.230	0.033	48.934	1	<0.001	1.258	(1.180, 1.342)
SSRS scores	-0.050	0.015	10.843	1	0.001	0.951	(0.923, 0.980)
PHQ-9 scores	0.108	0.033	11.037	1	0.001	1.115	(1.045, 1.188)
PHQ-15 scores	0.399	0.050	62.609	1	0.000	1.490	(1.350, 1.645)

PSQI, Pittsburgh Sleep Quality Index; SSRS, Social Support Rating Scale; PHQ-9, Patient Health Questionnaire-9; PHQ-15, Patient Health Questionnaire-15.

### Correlation between independent factors and varying levels of anxiety before gastrointestinal endoscopy

**Figure 1:**
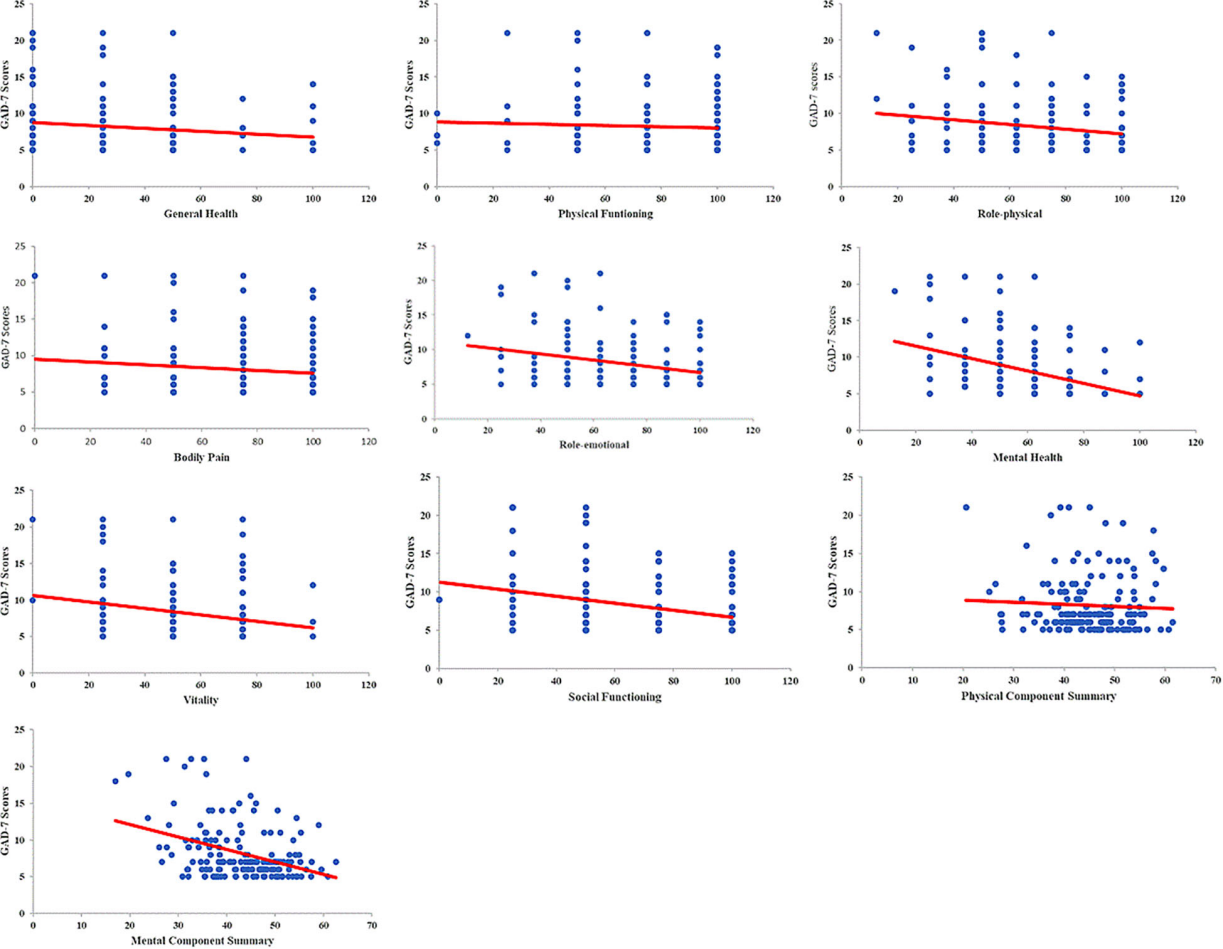
Correlation between QoL and varying levels of anxiety before gastrointestinal endoscopy. Scores among multiple domains of QoL, including GH, RP, RE, MH, VT, SF, and the scores of components in MCS, were negatively correlated with varying levels of anxiety among patients undergoing gastrointestinal endoscopy (*r* value were -0.164, -0.167, -0.192, -0.362, -0.216, -0.212 and -0.313, respectively; all *P*<0.05). There were no statistically significant correlations between anxiety levels among PF, BP and PCS (*r* value were -0.065, -0.049 and -0.039, respectively; all *P*>0.05).

This study also demonstrated that age, PSQI scores, PHQ-9 scores, and PHQ-15 scores were all positively correlated with varying levels of anxiety among patients who underwent gastrointestinal endoscopy. The correlation coefficients (*r*) were -1.51, 0.173, 0.466, and 0.407, respectively, with corresponding *P* values of 0.001, 0.026, <0.001, and <0.001. However, there were no statistically significant correlations between anxiety levels and SSRS scores (*r*= -0.047, *P*= 0.448) (**Figure 1**).

### Anxiety before gastrointestinal endoscopy and QoL

Patients who experienced anxiety prior to gastrointestinal endoscopy showed significantly lower QoL scores. All domains of QoL, including GH, PF, RP, BP, RE, MH, VT, SF, as well as the summary components PCS and MCS, were significantly reduced in patients with anxiety compared to those without (all *P*<0.05) (**Table 5**).

**Table 5:** Effect of anxiety before gastrointestinal endoscopy on quality of life.

Quality of life	Anxiety	Without anxiety	Statistical value
GH	30.42 ± 25.539	45.09 ± 23.479	*t*=6.324, *P*<0.001
PF	78.61 ± 25.712	85.21 ± 22.376	*t*=2.917, *P*=0.004
RP	68.60 ± 22.894	75.32 ± 23.861	*t*=3.016, *P*=0.003
BP	70.48 ± 22.911	82.67 ± 20.569	*t*=5.747, *P*<0.001
RE	66.87 ± 20.591	78.01 ± 21.149	*t*=5.591, *P*<0.001
MH	58.96 ± 16.958	73.89 ± 13.971	*t*=10.340, *P*<0.001
VT	54.97 ± 19.113	68.04 ± 19.288	*t*=7.110, *P*<0.001
SF	68.07 ± 24.406	83.07 ± 23.615	*t*=6.482, *P*<0.001
PCS	45.06 ± 7.573	47.58 ± 6.623	*t*=3.631, *P*<0.001
MCS	43.26 ± 8.486	50.43 ± 7.294	*t*=9.679, *P*<0.001

GH, General Health; PF, Physical Functioning; RP, Role-physical; BP, Bodily Pain; RE, Role-emotional; MH, Mental Health; VT, Vitality; SF, Social Functioning; PCS, Physical Component Summary; MCS, Mental Component Summary.

### Correlation between QoL and varying levels of anxiety before gastrointestinal endoscopy

**Figure 2:**
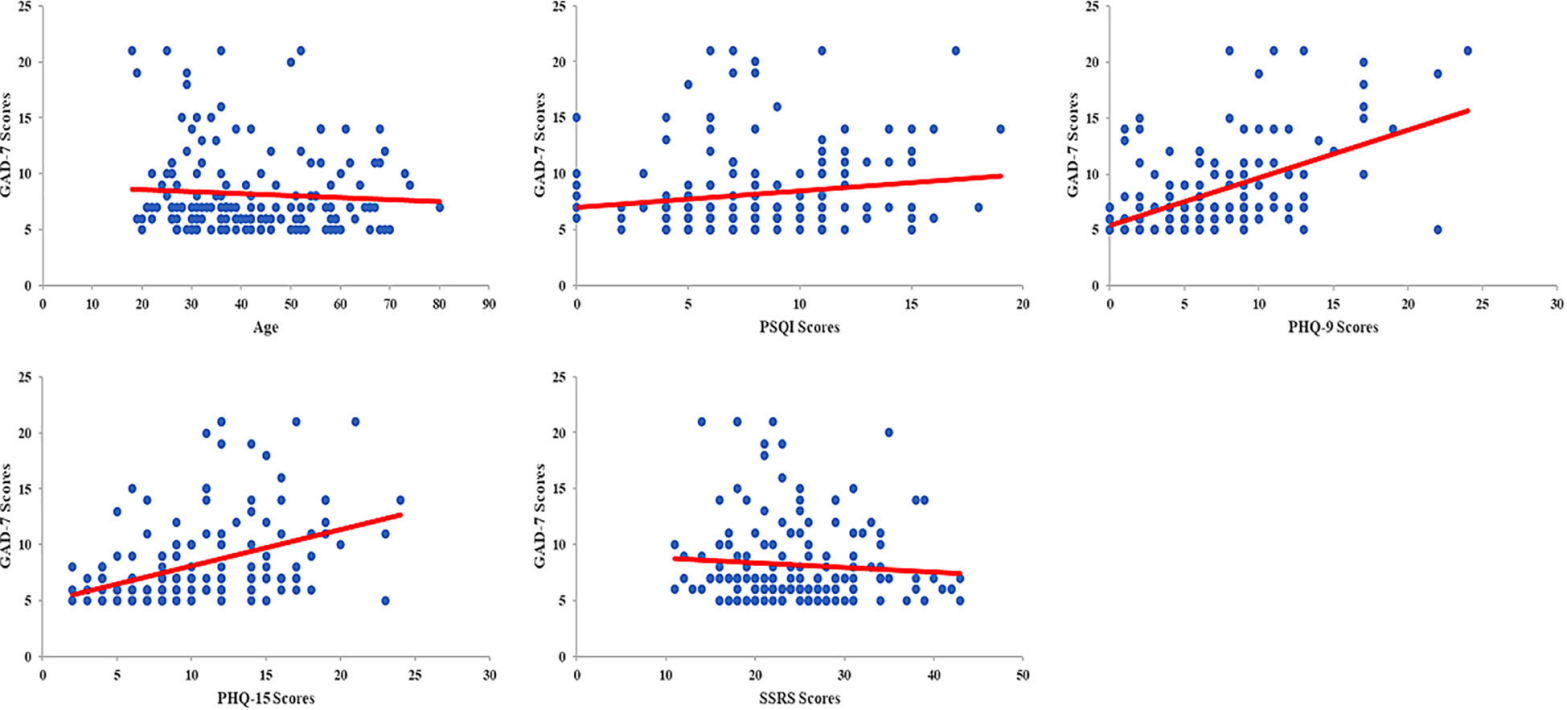
Correlation between independent factors and varying levels of anxiety before gastrointestinal endoscopy. Age, PSQI scores, PHQ-9 scores, and PHQ-15 scores were all positively correlated with varying levels of anxiety among patients who underwent gastrointestinal endoscopy (*r* value were -1.51, 0.173, 0.466, and 0.407, respectively; all *P*<0.05). There were no statistically significant correlations between anxiety levels and SSRS scores (*r*=-0.047, *P*= 0.448).

Scores among multiple domains of QoL, including GH, RP, RE, MH, VT, SF, and the scores of components in MCS, were negatively correlated with varying levels of anxiety among patients undergoing gastrointestinal endoscopy (all *P*<0.05). The more severe the anxiety, the lower the QoL scores observed (**Figure 2**).

## Discussion

Patients preparing to undergo invasive medical procedures such as gastrointestinal endoscopy frequently experience psychological distress, most commonly in the form of anxiety (29, 30). Globally, anxiety before gastrointestinal endoscopy is highly prevalent. Previous studies have reported rates ranging from 21.52% to over 50% among patients undergoing these procedures (17, 31). In this study, 34.44% of patients experienced anxiety before gastrointestinal endoscopy. Pre-procedural anxiety may reduce patient compliance and increase the risk of adverse events during or after the procedure (32). Therefore, identifying the contributing factors to this anxiety is critical to improving clinical outcomes.

Social-psychological factors are recognized as important pathogenic contributors to a wide range of diseases, particularly mental and mood disorders, such as bipolar disorder and suicidal behavior (33, 34). According to TOUS, psychological and environmental factors may induce the onset of unpleasant symptoms, including anxiety (12, 13, 35). In this study, we have confirmed that social-psychological factors such as social support, depression, and somatization had a significant impact on anxiety levels before gastrointestinal endoscopy. Furthermore, patients with unhealthy lifestyles, particularly those experiencing poor sleep quality, were more likely to suffer from pre-procedural anxiety. Recent research has suggested that sleep quality is closely associated with anxiety, potentially through mechanisms involving altered function of GABAergic neurons in the bed nucleus of the stria terminalis (36, 37).

According to the TOUS, physiological, psychological, and environmental factors all significantly contribute to the occurrence of unpleasant symptoms (12, 13, 35). In this study, we demonstrated that gender and age influenced the incidence of anxiety before undergoing gastrointestinal endoscopy. Differentiation of gender roles, disadvantage and disempowerment across the life course, and the coping styles of female that have an impact on female who was easy to suffer from anxiety (38). For the young age participants who underwent gastrointestinal endoscopy that were within negative health-related behaviors and would base on a false assumption with life-limiting conditions, that all would induce varying levels of anxiety (39, 40). However, other physiological and endoscopy-related factors, including family history of digestive diseases, H. pylori infection, number of previous endoscopies, use of sedation, type of endoscopic procedure, and diagnostic outcomes, did not show a significant impact on anxiety in this context, it may due to the sample homogeneity among participants. Anxiety, as a negative mood state, can be influenced by a range of physiological, psychological, and environmental factors (41–43). Additionally, individual differences in physiological, psychological, and social-environmental factors may shape one’s cognitive appraisal of invasive medical procedures. These differences in perceived acceptability may, in turn, lead to pre-procedural anxiety (44, 45). In this study, we identified gender, age, sleep quality, social support, depression, and somatization as independent predictors of anxiety before gastrointestinal endoscopy. Furthermore, PSQI, PHQ-9, and PHQ-15 scores were all positively correlated with varying levels of anxiety, confirming their relevance in evaluating psychological distress in this patient population.

Meanwhile, patients who experienced anxiety before undergoing gastrointestinal endoscopy also showed decreased QoL, representing one of the adverse outcomes associated with unpleasant symptoms (12, 13, 35). In this study, we confirmed that all QoL domains, both physical and mental components, were significantly lower in patients with anxiety. In particular, domains such as GH, RP, RE, MH, VT, SF, and MCS were negatively correlated with varying levels of anxiety prior to gastrointestinal endoscopy. Several studies have similarly reported that anxiety symptoms may impair physical functioning and that they are strongly associated with reduced QoL, especially in the physical dimensions. Moreover, as a negative mood state, anxiety tends to exert a more pronounced impact on the mental dimensions of QoL (46–48).

This study had several limitations. First, as a self-reported questionnaire-based survey, there is a possibility of memory bias or inaccuracies in the data provided by participants. Second, the study was conducted at a single center with a limited sample size, which may affect the generalizability of the findings. Further multicenter studies with larger samples are needed to validate these results.

In conclusion, this study confirmed a high prevalence of anxiety before gastrointestinal endoscopy. Socio-psychological factors played a significant role in the development of pre-procedural anxiety. Additionally, patients experiencing anxiety prior to endoscopy demonstrated reduced quality of life. These findings suggest that endoscopists, gastroenterologists, and GI nurses should pay close attention to patients at risk of anxiety before endoscopy in order to prevent adverse procedural outcomes and promote better recovery.

## Data Availability

The original contributions presented in the study are included in the article/supplementary material. Further inquiries can be directed to the corresponding author.
